# Tick-Borne Diseases in Sub-Saharan Africa: A Systematic Review of Pathogens, Research Focus, and Implications for Public Health

**DOI:** 10.3390/pathogens13080697

**Published:** 2024-08-17

**Authors:** Tidjani A. Djiman, Abel S. Biguezoton, Claude Saegerman

**Affiliations:** 1 Research Unit of Epidemiology and Risk Analysis Applied to Veterinary Sciences (UREAR-ULiège), Fundamental and Applied Research for Animals and Health (FARAH) Center, Faculty of Veterinary Medicine, University of Liege, 4000 Liège, Belgium; tidjaniayole.djiman@uliege.be; 2 Vector-Borne Diseases and Biodiversity Unit (UMaVeB), International Research and Development Centre on Livestock in Sub-humid Areas (CIRDES), Bobo-Dioulasso 454, Burkina Faso; babels005@yahoo.fr

**Keywords:** sub-Sahara Africa, tick-borne diseases, systematic review, pathogens, research, public health

## Abstract

Sub-Saharan Africa, with its hot and humid climate, is a conducive zone for tick proliferation. These vectors pose a major challenge to both animal and human health in the region. However, despite the relevance of emerging diseases and evidence of tick-borne disease emergence, very few studies have been dedicated to investigating zoonotic pathogens transmitted by ticks in this area. To raise awareness of the risks of tick-borne zoonotic diseases in sub-Saharan Africa, and to define a direction for future research, this systematic review considers the trends of research on tick-borne bacteria, parasites, and viruses from 2012 to 2023, aiming to highlight the circulation of these pathogens in ticks, cattle, sheep, goats, and humans. For this purpose, three international databases were screened to select 159 papers fitting designed inclusion criteria and used for qualitative analyses. Analysis of these studies revealed a high diversity of tick-borne pathogens in sub-Saharan Africa, with a total of 37 bacterial species, 27 parasite species, and 14 viruses identified. Among these, 27% were zoonotic pathogens, yet only 11 studies investigated their presence in humans. Furthermore, there is growing interest in the investigation of bacteria and parasites in both ticks and ruminants. However, research into viruses is limited and has only received notable interest from 2021 onwards. While studies on the detection of bacteria, including those of medical interest, have focused on ticks, little consideration has been given to these vectors in studies of parasites circulation. Regarding the limited focus on zoonotic pathogens transmitted by ticks, particularly in humans, despite documented cases of emerging zoonoses and the notable 27% proportion reported, further efforts should be made to fill these gaps. Future studies should prioritize the investigation of zoonotic pathogens, especially viruses, which represent the primary emerging threats, by adopting a One Health approach. This will enhance the understanding of their circulation and impact on both human and animal health. In addition, more attention should be given to the risk factors/drivers associated to their emergence as well as the perception of the population at risk of infection from these zoonotic pathogens.

## 1. Introduction

Ticks are hematophagous arthropods found worldwide, from desert areas to polar regions. Approximately 900 species of tick have been identified, with 700 belonging to the Ixodidae family (hard ticks) and 200 to the Argasidae family (soft ticks) [1]. Ticks are primarily ectoparasites of wild vertebrates. However, through contact with wild fauna, some species have adapted to domestic or livestock animals, particularly cattle and small ruminants, resulting in a significant economic impact [2]. Rural populations in tropical areas are most affected due to factors favouring tick proliferation and the extensive livestock farming system that ensures contact with wildlife and tick dissemination [2,3].

As exclusive ectoparasites of vertebrates, ticks can become infected while feeding and transmit a wide variety of bacteria (e.g., spirochetes, *Rickettsia*), parasites (e.g., *Babesia*, *Theileria*), and viruses (e.g., flaviviruses, nairoviruses) to their host [4]. Moreover, a single species, depending on its life cycle, can infest a range of hosts during its life cycle, from wild animals to domestic animals and humans. Thus, ticks play a significant role in species barrier crossing and are implicated in various animal and zoonotic diseases. They are responsible for viral infections such as Crimean-Congo haemorrhagic fever, West Nile fever, Omsk haemorrhagic fever, and Colorado tick fever; bacterial infections such as Q fever, Lyme disease, relapsing fever borreliosis, as well as animal borrelioses; and protozoal infections such as theileriosis and babesiosis [1]. The majority of tick-borne infections are zoonotic, and their incidence and distribution are steadily increasing worldwide [5,6,7]. In Europe, the incidence of Lyme borreliosis in 2018 was estimated to range from 1 to 365 cases per 100,000 person-years [8]. Meanwhile, in 2022, the United States reported a total of 62,551 cases of Lyme borreliosis [9]. In addition, several cases of emergence have been reported. For example, severe fever with thrombocytopenia syndrome has been identified in China in 2009, and Heartland virus and Bourbon virus in the United States, respectively, in 2012 and 2014 [10,11,12]. These emergence cases are added to the re-emergence and geographical expansion of Crimean-Congo haemorrhagic fever and tick-borne encephalitis [6,13].

Economic losses associated with tick management and treatment of tick-borne infections are estimated at nearly USD 20 billion annually in developing countries [14]. In these parts of the world, besides losses due to reduced milk and meat production, over three million animals (mostly non-tropical breeds) are reported dead from tick-borne infections every year [14].

In sub-Saharan Africa (SSA), ticks and the diseases they transmit pose a major challenge to animal and human health. Indeed, with its climate (hot and humid) favourable to tick proliferation, this region of Africa harbours a wide variety of ticks species capable of infesting both animals and humans [15]. In addition, in this region of Africa, the livestock farming system is generally low-input and based on the exploitation of natural resources through internal and transboundary transhumance [16,17]. This leads to the exposure of animals and pastoralists to tick-prone habitats and the dissemination of tick species and the pathogens they transmit. Additionally, the invasion of new species and the emergence of the pathogens they transmit have been observed [18]. The most notable is the incidental introduction of the *R. microplus* tick, an invasion accompanied by the appearance of several foci of babesiosis due to *B. bovis*, of which it is the vector [19].

More than nine genera of tick are found in SSA, with a high burden of different species of the genera *Amblyomma*, *Hyalomma* and *Rhipicephalus* involved in the epidemiology of various zoonotic and non-zoonotic diseases such as rickettsiosis, ehrlichiosis, babesiosis, borreliosis, and anaplasmosis, and viruses including Dugbe virus, Bhanja virus, and Crimean-Congo haemorrhagic fever virus [20].

Despite the increasing presence of tick species vectors of zoonotic pathogens in SSA [19,21,22,23,24,25], evidence of the emergence and re-emergence of tick-borne pathogens, and the circulation of agents responsible for zoonotic diseases, there is still a low research dynamic on tick-borne zoonotic diseases [26,27,28]. Through this systematic review, the research dynamic on the epidemiology of tick-borne diseases (TBDs) in SSA is highlighted to provide an overview of the circulation of tick-borne pathogenic agents, and to discuss the implications of these results for public health and future scientific research.

To better understand the interactions related to pathogen circulation in the area, we will consider prevalence studies conducted on ticks, livestock, and humans from 2012 to 2023. Cattle, sheep, and goats are the three most representative species of livestock in SSA, collectively accounting for 88% of the total livestock population in the region [29]. These three species will constitute the animal populations in this review. Regarding the pathogens, we will explore studies on bacteria, parasites, and viruses transmitted by ticks in each targeted population. Thus, we will exhaustively determine the extent of research conducted for the detection of each group of pathogens and compare the research dynamic between these groups.

Moreover, the efficiency and accessibility of diagnostic techniques are crucial in detecting pathogens. Indeed, due to their effectiveness, evidence of technique mastery, and widespread use of these techniques in the area, we limit ourselves to studies that have used molecular techniques, specifically polymerase chain reaction (PCR) and its derivatives. For its exploratory nature, interest will also be given to papers that have used metagenomics.

## 2. Materials and Methods

### 2.1. Research Question and Applied Formula

This review was performed following the PRISMA guideline (Table S1 in Supplementary Materials). The research questions are defined using a Population, Intervention, Comparison, and Outcome (PICO) approach. More precisely, the target populations are represented by ticks, cattle, sheep, goats, and humans living in SSA. The chosen diagnosis methods were the molecular (PCR and reverse line blotting (RLB)) and the genomic (metagenomic) tests. Unfortunately, a comparison of the results between these two methods was not available. The outcome was the presence or absence of tick-borne bacteria, parasites, or viruses. Following these PICO components, the following research questions have been formulated: How does an interest in research on each tick-borne pathogen domains relate to the target populations in SSA? Which tick pathogen species have been detected in ticks, cattle, sheep, goats, and humans in SSA? What is the distribution of these pathogens? Which laboratory test are most often used to detect each class of these pathogens? To address these questions, three databases, i.e., PubMed, Scopus, and ScienceDirect, were examined. The following basic search formula was designed and adapted to each database: (Africa) AND (tick OR human OR cattle OR sheep OR goat) AND (anaplasma OR rickettsia OR ehrlichia OR coxiella OR wolbachia OR borrelia OR babesia OR theileria OR virus). The search equation used for each database was constructed to include free-text terms, keywords (in the title, abstract, or author keywords) and any appropriate subject indexing (e.g., MeSH in pubMed) (Table 1).

### 2.2. Eligible Criteria, Article Screening, and Data Extraction

The papers included in this review are restricted to (I) original articles; (II) articles written in either English or French; (III) reported cross-sectional studies; (IV) published studies using ticks or blood collected from humans, cattle, sheep, and goats in SSA; (V) published studies using PCR or any variant of PCR, RLB, and metagenomic tests; (VI) published studies aiming to detect the presence or absence of tick-borne bacteria, protozoa, and viruses in target populations. Papers not based on the PICO components: publications related to poster sessions, interviews, abstracts, symposia, oral presentations and reviews, as well as unavailable full texts or abstract-only papers were excluded.

The databases were interrogated from 25 July 2023 to 7 September 2023. Based on the recent paper of Cossu et al., (2023) [30], which evidenced a high interest in tick pathogens in Africa from 2012, the results of each database interrogated were filtered to select just those from 2012 to 2023. The retained references were exported from the databases and imported in the same file in Zotero manager. In Zotero, firstly, duplications were deleted. Then, based on the defined eligible criteria, articles were screened by reading their title and abstract to determine whether they were relevant to the research question. Two independent reviewers performed this screening step. The full texts of the selected articles were retrieved when they include data on tick-borne pathogen prevalence and screened with regards to the eligible criteria.

Six categories of data were extracted: target populations, pathogens, diagnosis tests, prevalence, sampling, and study design.

### 2.3. Data Analysis

The Excel raw data containing the data extracted from the articles were imported into R studio software (version 4.3.3) sourced from Boston, MA, USA, for qualitative analysis. The relationship between pathogens and variables, such as the number of studies, countries, and target populations, was assessed using descriptive statistics and illustrated through tables, bar plots, and choropleth maps. This approach allows for an appreciation of trends in tick-borne pathogen research, highlights the most frequently reported pathogens in each target population, identifies the most commonly used diagnostic methods, and depicts the distribution of these pathogens across SSA.

## 3. Results

### 3.1. Overview on Screened Articles and Relevance of Pathogens in Targeted Populations

**Figure 1:**
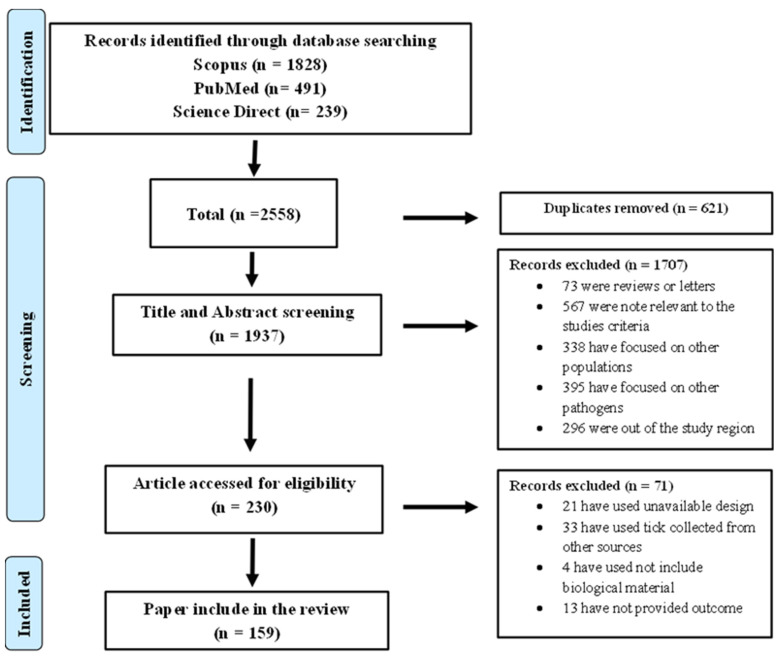
PRISMA flow diagram.

A total of 2558 potentially relevant articles were found. After removing duplicates, 1937 papers were screened by title and abstract (Figure 1). The 230 remaining references after title and abstract screening were subject to full text screening. Based on this selection process and the defined eligibility criteria, a total of 159 original papers were retained for the systematic literature review.

**Figure 2:**
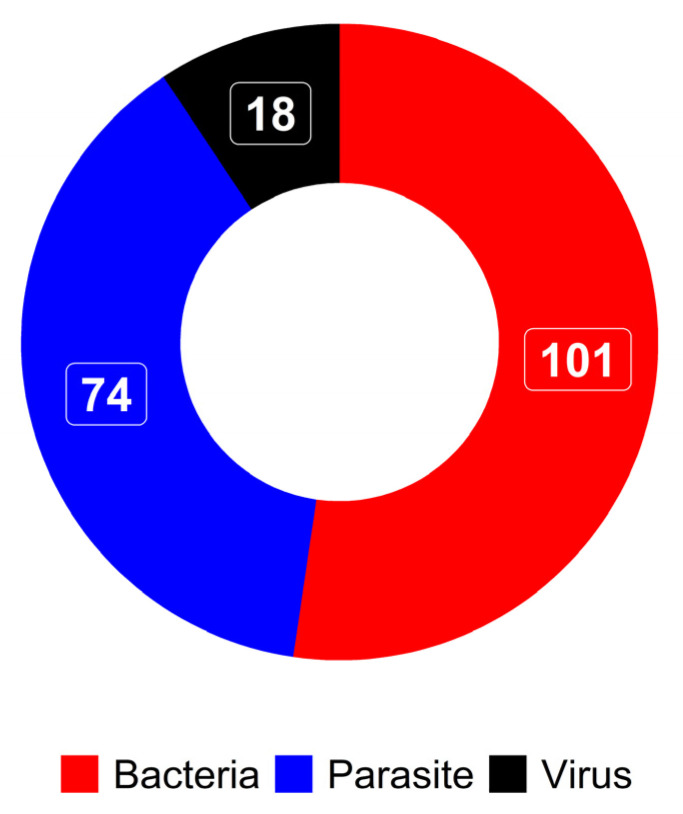
Number of studies according to types of pathogens.

**Figure 3:**
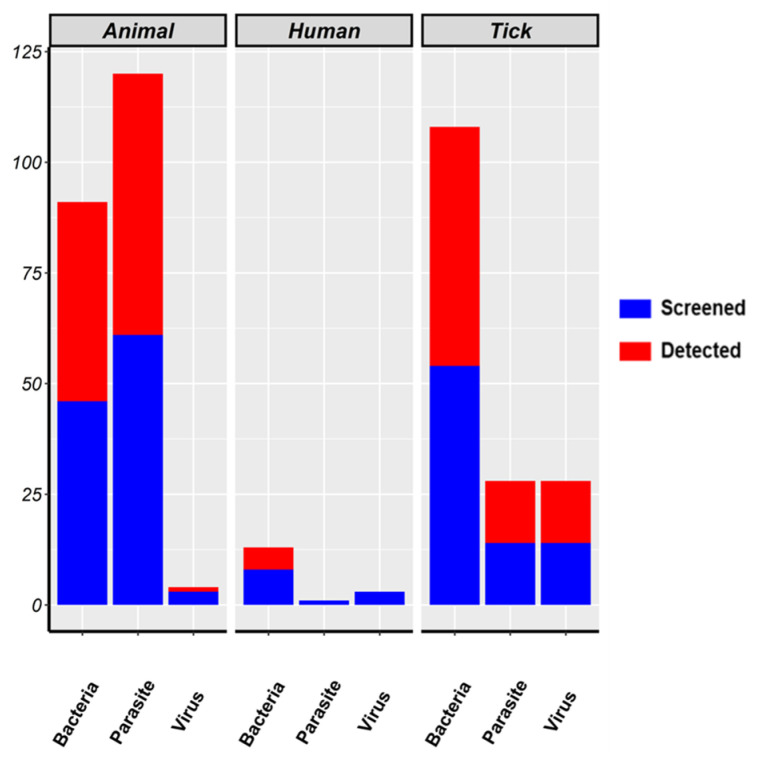
Pathogens studies according to target population. Legend: “Screened” refers to the total number of studies that investigated the presence of each pathogen, regardless of whether the pathogen was detected or not. “Detected” indicates the number of studies in which the pathogen was actually detected.

Most studies focus on bacteria (N = 101), then parasites (N = 74) and viruses (N = 18) (Figure 2). Bacteria were primarily screened in ticks, followed by animals, and then humans. Parasites were mostly screened in animals, followed by ticks, and then humans. In humans, bacteria were the most investigated. The proportion of detection for each type of pathogen in the target populations follows the same trend (Figure 3).

### 3.2. Temporal Evolution of the Selected Papers’ Interests

**Figure 4:**
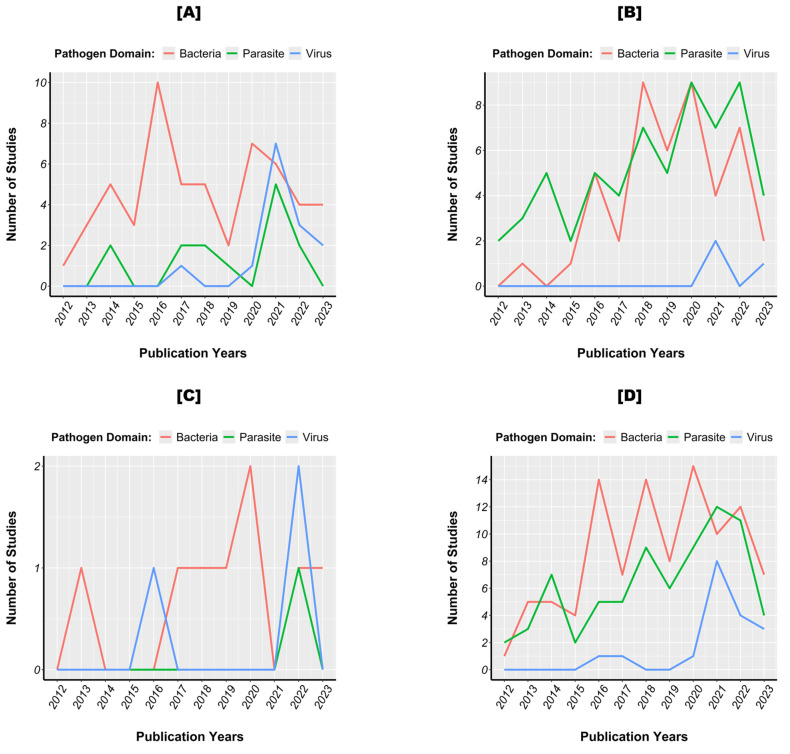
Temporal evolution of studies on tick (**A**), animal (**B**), human (**C**), and combined (**D**) target populations.

Figure 4 illustrates the temporal evolution of studies across the target populations, highlighting an increasing focus on the detection of bacteria and parasites in animals. Viral screenings in animals occurred in both 2021 and 2023. For ticks, research has consistently focused on bacteria, with a notable increase in interest for parasites and viruses in 2021. Human studies showed no clear trend, with a maximum of two papers each on bacteria and viruses and one on parasites. Despite specific tendencies within each target population, there has been a general surge in research interest in tick-borne bacteria, parasites, and viruses, with bacterial studies leading, followed by those on parasites and viruses (Figure 4D).

### 3.3. Relationship between Screened and Detected Pathogen Species in Target Populations

**Figure 5:**
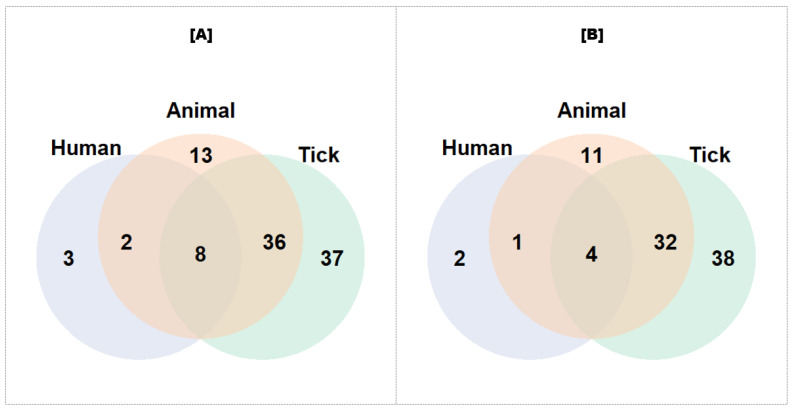
Venn diagram of screened (**A**) and detected (**B**) pathogens in animals, ticks, and humans. Legend: “Screened” refers to the total number of studies that investigated the presence of each pathogen, regardless of whether the pathogen was detected or not. “Detected” indicates the number of studies in which the pathogen was actually detected.

Out of the 99 tested, a total of 88 (including confirmed and candidate species) tick-borne pathogens were detected in humans, cattle, sheep, goats, and their ticks in SSA (Figure 5). Of the 81 pathogenic species sought in ticks, 74 were found, while 48 out of 59 pathogenic species were detected in animals. However, only 7 out of the 13 pathogenic species screened from human blood samples were detected. Ticks carried 38 exclusive pathogens, while 11 were exclusively found in animals, and two were exclusively found in humans. The three target populations shared four pathogens. However, ticks and animals shared 36 pathogens, while five pathogens were shared by humans and animals. All of the pathogens shared by ticks and humans are reported in animals. When comparing the proportion of detection/screening between ticks and animals and between animals and humans, using the Bonferroni correction, it was found to be equal with *p*-values of 0.4 and 0.23, respectively. However, the proportion was found to be different between ticks and humans, with a *p*-value of 0.04. In addition, the Spearman correlation coefficient between screened and detected pathogens was significantly positive in both ticks (0.54) and animals (0.92), with *p*-values of 3.7 × 10^−7^ and 2.2 × 10^−16^, respectively. However, this correlation was not significant in humans (*p* = 0.32).

### 3.4. Pathogens Detected According to Populations: Highlighting Tick-Borne Zoonotic Pathogens

In this section, we present the key findings of the systematic review on the microorganisms infecting livestock, ticks, and humans in SSA. To enhance readability while maintaining comprehensive documentation, the references supporting the observations presented in this chapter have been collated in Table A1. Readers are encouraged to consult the Table A1 and Supplementary File S1 for detailed information on individual studies. For particularly points, references will be provided directly in the text.

Livestock (i.e., cattle, sheep, and goats), ticks, and humans in SSA are susceptible to infection by a variety of microorganisms. These microorganisms include 15 species of *Anaplasma* and eight species of *Ehrlichia* from the Anaplasmataceae family; *Coxiella burnetii* from the Coxiellaceae family; eight species of *Rickettsia* and one species of *Wolbachia* from the Rickettsiaceae family; and five species of *Borrelia*. This classification encompasses both confirmed species and those with candidate status. Additionally, there are 13 species of *Babesia* and 15 species of *Theileria* which are classified as parasites. Fifteen viruses have been reported, with eight belonging to the Bunyaviridae family, one to the Flaviviridae family, two to the Nairoviridae family, one to the Peribunyaviridae family, two to the Poxviridae family, and one to the Togaviridae family. None of the viruses were detected in humans [31,32,33], and only one Kaptombes virus (KPTV) was identified in animals. All of these viruses, except for KPTV, have been reported in ticks, with a high frequency of Crimean-Congo haemorrhagic fever virus (CCHFV) (six studies).

In animal populations, studies have reported *Anaplasma marginale* and *Ehrlichia ruminantium* 31 and 20 times, respectively. *Anaplasma centrale* (11 studies), *Anaplasma platys-Like* (11 studies), and *Anaplasma ovis* (11 studies) were also frequently reported. Ticks were most commonly investigated for *E. ruminantium* (12 studies), *A. marginale* (10 studies), and *Ehrlichia canis* (six studies) within the Anaplasmatacea family. *Hyalomma truncatum*, *Rhipicephalus decoloratus*, and four species belonging to the genus *Amblyomma* (*A. hebraeum*, *A. gema*, *A. coherence*, *A. variegatum*) have been found to carry *E. ruminantium*. *A. hebraeum*, *A. variegatum*, *Rhipicephalus evertsi*, *Rhipicephalus sanguineus*, and *H. truncatum* have been detected carrying *E. canis*. *A. marginale* has been reported in two tick genera. In humans, *Anaplasma phagocytophilum* was screened and detected by only one study [34].

Among the Anaplasmataceae reported, *A. marginale*, *E. ruminatium*, and *A. centrale* are the most commonly distributed in SSA countries, with 17, 16, and 14 countries, respectively. *A. ovis*, *A. Platys-Like*, and *E. canis* are reported to be present in nine, seven, and five different SSA countries, respectively. Additionally, five zoonotic agents of Anaplasmatacea have been found in ticks: *Anaplasma capra*, *A. platys-Like* (reported in *Rhipicephalus* ticks), *A. phagocytophylum*, *Ehrlichia chaffeensis* (detected in *A. hebraeum*), and *E. canis*. *A. capra* was detected in pooled ticks of *Rhipicephalus* spp. while *A. platys-Like* was detected in *R. evertsi* and *Rhipicephalus microplus*. *A. phagocytophilum* has been reported in *Amblyomma* species such as *A. variegatum*, *A. coharensis*, *A. hebraeum,* and *A. lepidium*. *A. phagocytophilum* has also been detected in cattle, sheep, and humans. *A. platys-Like* was found in the all three animal populations considered. Both *E. chaffeensis* and *E. canis* were found in cattle, while *E. canis* was also found in goats.

Besides the candidatus species, all known and reported Rickettsiacea are zoonotic. *Rickettsia africae* was the most commonly detected. It has been found in ticks from the genera *Amblyomma* (*A. cohaerens*, *A. gemma*, *A. hebraeum*, *A. lepidum*, *A. variegatum*), *Hyalomma* (*H. impressum*, *H. marginatum*, *H. truncatum*), and *Rhipicephalus* (*R. annulatus*, *R. appendiculatus*, *R. decoloratus*, *R. evertsi*, *R. microplus*, *R. sanguineus*). In animals, *Rickettsia africae* and *Rickettsia felis* have been found in only one study. Although *Rickettsia felis* and *Rickettsia bellili* have not been sought in ticks, one study has reported their occurrence in humans. Among Coxiellaceae, *C. burnetti*, which is known to be zoonotic, was the only species detected and the most prevalent bacterium studied in humans. It has been reported in eight countries in SSA. Eleven studies reported its presence in ticks, one in animals, and two in humans. *Borrelia theileri* and *Borrelia crocidurae* (zoonotic) were the known pathogens of the genus *Borrelia* that were reported. *B. theileri* was identified in a pool of *Rhipicephalus geigyi*/*R. decoloratus* collected in Mali and in blood samples from cattle in Cameroon, while *B. crocidurae* was only screened and detected in a human sample from Senegal [35].

### 3.5. Tick Genera and Pathogen Family

**Figure 6:**
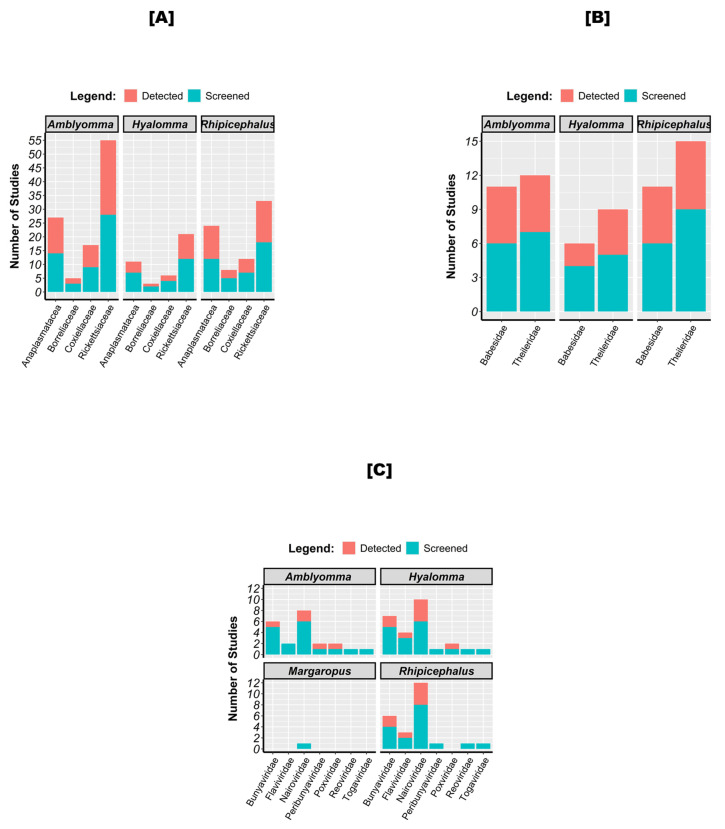
Bacteria (**A**), parasite (**B**), and virus (**C**) families according to tick genus. Legend: “Screened” refers to the total number of studies that investigated the presence of each pathogen, regardless of whether the pathogen was detected or not. “Detected” indicates the number of studies in which the pathogen was actually detected.

Three main tick genera have been studied in SSA for the detection of tick-borne pathogens (Figure 6): *Amblyomma*, *Hyalomma*, and *Rhipicephalus* (including *Boophilus*). Among the tick-borne bacteria, Rickettsiacea was mostly screened in each target tick genus, followed, respectively, by Anaplasmatatcea, Coxiellacea, and Borreliacea. Except for the latter, which was most commonly screened in *Rhipicephalus*, *Amblyomma* has been better studied for detecting all pathogen families, followed by *Rhipicephalus*. Regarding the parasites, Theileridae was more frequently reported than Babesidae in each tick genus, with a higher prevalence in *Rhipicephalus* followed by *Hyalomma*. In *Amblyomma*, both parasite families were equally prevalent. For viruses, Nairoviridae was the most commonly detected, followed by Bunyaviridae and Flaviviridae. Nairoviridae was detected in four out of eight studies in *Rhipicephalus*, four out of six studies in *Hyalomma*, and two out of six studies in *Amblyomma*. Unlike other genera, Flaviviridae was not detected in *Amblyomma*.

### 3.6. Method Used Most to Detect Bacteria, Parasites, and Viruses in the Target Population

**Figure 7:**
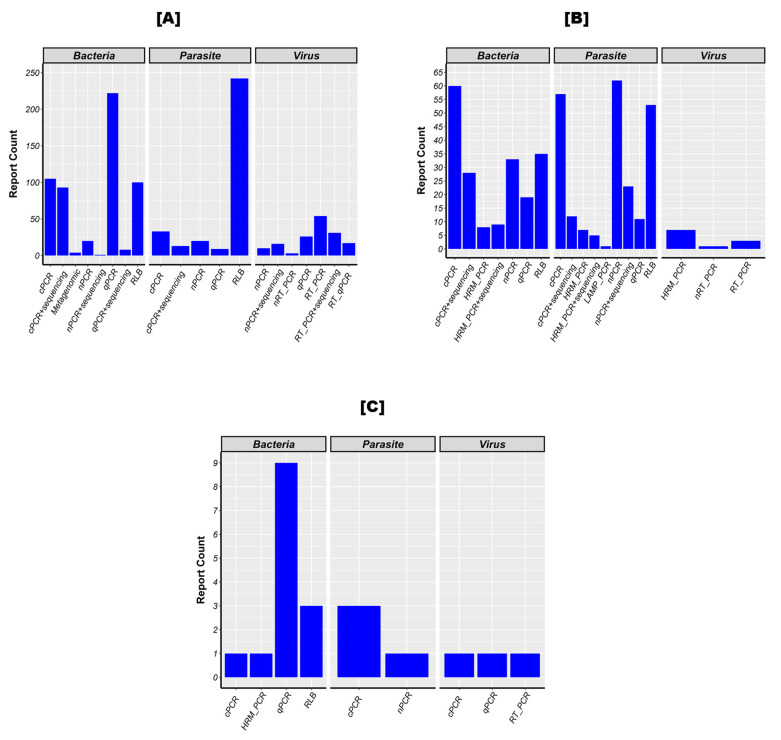
Methods used to detect a domain’s pathogens in ticks (**A**), animals (**B**), and humans (**C**). Legend: This figure represents the number of times each method has been used to detect pathogens belonging to each pathogen domain (bacteria, parasites, and viruses) in ticks (**A**), animals (**B**), and humans (**C**). The various methods include the following: cPCR: conventional polymerase chain reaction; cPCR+sequencing: conventional polymerase chain reaction followed by the sequencing of the positive amplicons; nPCR: nested polymerase chain reaction; nPCR+sequencing: nested polymerase chain reaction followed by the sequencing of the positive amplicons; qPCR: quantitative polymerase chain reaction; qPCR+sequencing: quantitative polymerase chain reaction followed by the sequencing of the positive amplicons; RT_PCR: reverse transcription polymerase chain reaction; nRT_PCR: nested reverse transcription polymerase chain reaction; RT_PCR+sequencing: reverse transcription polymerase chain reaction followed by the sequencing of the positive amplicons; RT_qPCR: reverse transcription quantitative polymerase chain reaction; HRM_PCR: high-resolution melting polymerase chain reaction; HRM_PCR+sequencing: high-resolution melting polymerase chain reaction followed by the sequencing of the positive amplicons; LAMP: loop-mediated isothermal amplification; RLB: reverse line blot hybridization assay; and Metagenomic.

Tick-borne pathogens have been detected using various methods (Figure 7). Bacteria were mostly detected in ticks by quantitative polymerase chain reaction (qPCR), followed by conventional polymerase chain reaction (cPCR), reverse line blot hybridization assay (RLB), and cPCR + sequencing. RLB was most preferred to detect parasites, while reverse transcription polymerase chain reaction (RT-PCR) was reported to detect viruses.

When considering livestock populations, the preferred methods for detecting bacteria and parasites are cPCR, nested polymerase chain reaction (nPCR), and RLB. Regarding viruses, high-resolution melting polymerase chain reaction (HRM-PCR) and nested reverse transcription polymerase chain reaction (nRT-PCR) were used, but only RT-PCR was able to detect them. In humans, parasites were screened using cPCR and nPCR, while viruses were screened using cPCR, qPCR, and RT-PCR.

### 3.7. Tick-Borne Pathogen Distribution: Focus on Viruses and Zoonotic Bacteria and Parasites

**Figure 8:**
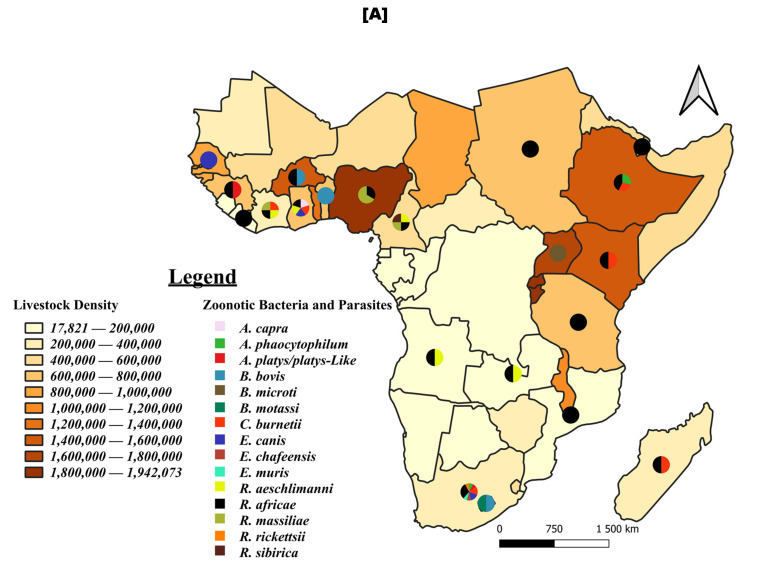

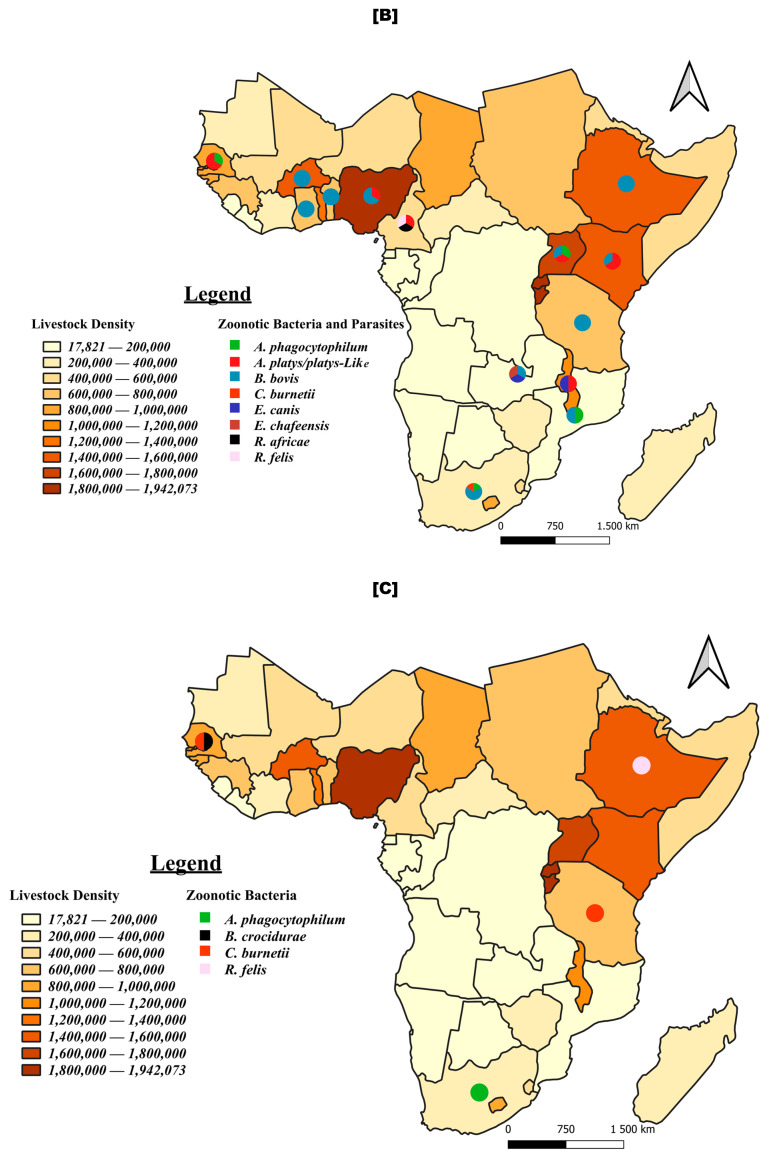
Distribution of tick-borne zoonotic bacteria and parasites in ticks (**A**), animals (**B**), and humans (**C**). Legend: These figures illustrate the geographical distribution and frequency of studies reporting the presence of zoonotic bacterial and parasitic agents transmitted by ticks in ticks (**A**), animals (**B**), and humans (**C**) in sub-Saharan Africa. Each pathogen is represented by a distinct color. The pie charts superimposed on the different countries indicate the frequency of studies reporting each pathogen in each country. The underlying map shows the average density of the ruminant population (cattle, sheep, goats) between 2012 and 2022, based on FAO statistics [36]. The density is expressed as the number of animals per square mile.

**Figure 9:**
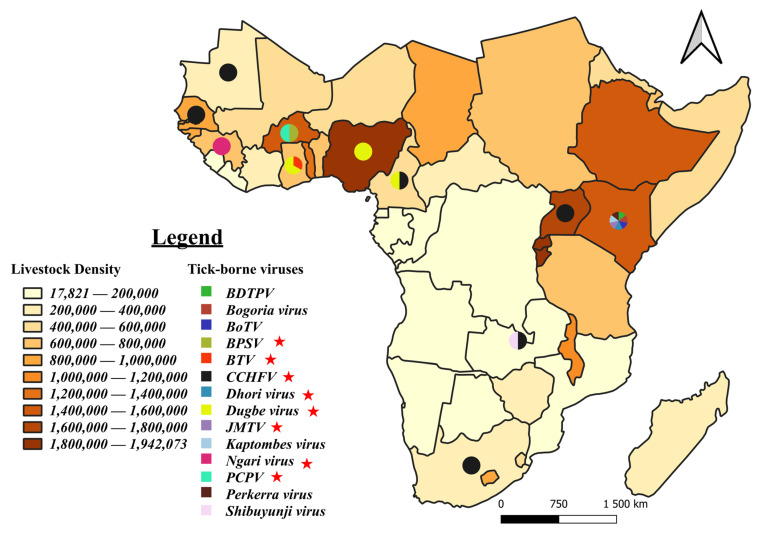
Distribution of tick-borne viruses. Legend: The figure illustrates the geographical distribution and frequency of studies reporting the presence of viruses transmitted by ticks in in sub-Saharan Africa. Each virus is represented by a distinct color. The pie charts superimposed on the different countries indicate the frequency of studies reporting each virus in each country. The underlying map shows the average density of the ruminant population (cattle, sheep, goats) between 2012 and 2022, based on FAO statistics [36]. The density is expressed as the number of animals per square mile. The red stars indicate a zoonotic virus; BDTPV: Brown dog tick phlebovirus; BOGV: Bogoria virus; PERV: Perkerra virus; JMTV: Jingmen tick virus; BPSV: Bovine papular stomatitis virus; PCPV: Pseudocowpox virus; KPTV: Kaptombes virus; BTV: Balanbala tick virus; BoTV: Bole tick virus.

Out of the 78 known pathogens, 22 have been identified as zoonotic. Figure 8 and Figure 9 illustrate their distribution in relation to livestock density.

Ticks were found to harbour 12 zoonotic bacteria and three zoonotic parasites, distributed in 20 countries, with a higher concentration where livestock density is at least 40,000 animals per square mile. South Africa reported 66.67% of these zoonotic bacteria, while Ghana, Ivory Coast, and Cameroon reported 5, 4, and 4, respectively. Parasites have been reported only in Benin, Burkina Faso, Lesotho, and Uganda. The most common zoonotic bacteria detected in ticks were *R. africae* (found in 17 countries) and *A. platys* (found in seven countries) (Figure 8A).

Livestock from 14 countries were found to be infected with seven different bacteria and one parasite, *Babesia bovis*, which was the most common pathogen in 11 out of the 14 countries. The bacteria *A. platys* and *A. phagocytophilum* were also commonly found, reported in six and four countries, respectively. Similar to ticks, these pathogens were predominantly reported in animals from the western part of the study area, followed by the eastern part. South Africa was the southernmost country where the pathogens were reported (Figure 8B).

Only bacteria were detected in humans. *A. phagocitophylum*, *C. burnetii*, and *R. felis*, which are present in ticks and animals, have also been reported in humans. These pathogens were reported in humans from South Africa, Tanzania, Ethiopia, and Senegal. *B. crocidurae* was the only zoonotic *Borrelia* species reported and was found exclusively in humans (Figure 8C).

Fourteen different viruses have been reported and distributed in 11 SSA countries (Figure 9). Eight of them have been identified as zoonotic. Kenya has been reported to host the most viruses, with a total of seven. No evidence has been found to suggest a relation between livestock density and the distribution of tick-borne viruses in comparison with bacteria and parasites.

## 4. Discussion

### 4.1. Tick-Borne Pathogen Research Focus and Implications for Public Health

Tick-borne diseases pose significant threats in tropical areas, particularly in sub-Saharan Africa (SSA), which is characterized by diverse tick species and the close interconnections between human, animal, and wildlife populations [2,37,38]. This systematic review demonstrates the overriding focus on bacterial pathogens (in contrast to viruses and parasites) transmitted by ticks in SSA. The differential attention to these pathogen groups is the product of a complex interplay of factors, such as disease prevalence and implications for public health needs. Tick-borne diseases (TBDs) resulting from bacteria are given priority as a public health issue over parasites and viruses, with the exception of some viruses which primarily affect humans and have recently gained increased attention. Among the well-documented TBDs in Africa, six are caused by bacteria (including tick-borne spotted fever, tick-borne relapsing fever, anaplasmosis, ehrlichiosis, bartonelosis, and Q fever), two by parasites (theileriosis and babesiosis), and two by viruses (Crimean-Congo hemorrhagic fever and African swine fever) [21,39,40,41,42]. Also, the distribution patterns of tick species across various SSA regions have further increased the focus on bacterial transmission by ticks. In particular, *A. variegatum* and *A. hebraeum*, among the most common tick species [20], are known for their capacity to transmit bacteria to both humans and animals more than for their transmission of parasites and viral pathogens [43,44,45,46]. This implication for common tick species in Africa based on a research preference for tick-borne bacterial infections is further supported by the significance of the screening of ticks for bacterial detection more than parasites and viruses (Figure 3). Research efforts predominantly target tick-borne bacteria in ticks, while tick-borne parasites are extensively studied in ruminants. It is noteworthy that the prevalence, spread, and associated risk factors of tick-borne bacteria has grown in importance due to their impact on animal and human health. Thus, knowing which tick species can act as vectors, surveillance and efforts to prevent their spread have become the subject of considerable research. However, the same pattern is not observed with surveillance based on the prevalence and incidence of parasites, particularly in domestic animals. Along these lines, implementation of preventive and control strategies for parasitic diseases is indeed prioritized. Numerous factors can explain this strategic focus. Examples include their high economic impact on livestock farming [47] and the evidence supporting the participation of common ticks in the epidemiology of parasitic diseases [21].

Regarding studies on the prevalence of tick-borne pathogens in humans, the focus was more on bacteria too. Nine bacterial species were investigated, compared to three parasites and one virus. These results confirm, as highlighted above, the importance attributed to tick-borne bacteria as a public health concern in this region of Africa.

Over time, studies have shown a marked change in interest in monitoring tick-borne pathogen agents. Initially, the focus was on the detection of bacteria and parasites. However, this trend has gradually evolved to also include a marked interest in viruses, as observed in the years 2021 and 2023. The shift towards the study of viruses can be associated to a growing concern about the emergence of viral diseases, which are directly linked to climate change and agricultural practices [48]. Indeed, several zoonotic infectious diseases caused by new tick-borne viruses have been reported worldwide in recent years. Examples include severe fever with thrombocytopenia syndrome identified in China in 2009, and the Heartland virus and Bourbon virus in the United States, respectively, in 2012 and 2014 [10,11,12]. In addition to these cases of emergence, re-emergence cases and the geographical expansion of Crimean-Congo haemorrhagic fever and tick-borne encephalitis, both discovered over 50 years ago, have been reported [6]. However, it is worth noting that the increased interest in viruses in Africa since 2021 appears to be catalyzed by the emergence of the COVID-19 pandemic. The emergence of this major disease has raised concerns about the dangers of viral diseases and led to a general sense of caution towards emerging diseases. The concurrent increase in attention to parasites during the same period (2021) illustrates this caution.

Furthermore, this study demonstrated the significant diversity of tick-borne pathogens among ticks, animals, and humans in SSA. Of the 99 infectious agents screened, 88 were identified, indicating a high detection success rate of 88.89%. Moreover, ticks, animals and humans carried respectively thirty-eight, eleven, and two pathogens, with four pathogens shared among all populations. Ticks and animals shared thirty-six pathogens, while humans and animals shared five. This highlights the central role that ticks play in the transmission and maintenance of pathogens within SSA agro-ecosystems [16]. The considerable overlap of ticks and livestock regarding pathogens clarifies the central role of tick-driven pathogen transmission in SSA agro-ecosystems. However, all tick-shared pathogens with humans were observed to also be present in animals, illustrating the zoonotic potential of infectious agents and livestock’s key role as a reservoir host for tick-borne zoonotic agents. Hence, increased research into risk factors associated with infectious agents’ transmission to humans will be crucial for the development of effective strategies to prevent and control TBDs.

Additionally, when the detection rates in ticks, animals, and humans were compared, there was a significant difference in those between ticks and humans, indicating that pathogens are not as common in this group as in the others. Interestingly, a positive correlation between the screening/detection of ticks and animals suggests the efficacy of reported detection methods used to detect pathogens in the samples from these target populations. In contrast, lack of such a correlation in humans raises questions about the efficacy of the methods used for pathogen detection, as well as their prevalence in this host group. It is evident that unlike ticks [49,50] and animals [51,52,53], humans are not a natural reservoir, and only pathogens with zoonotic potential can be detected in humans. Studies addressing pathogen detection in humans, such as those involved in this study, were conducted on patients with febrile illnesses, as fever is a major clinical sign of tick-borne zoonotic diseases. However, considering the results obtained, although the improvement of diagnostic techniques is debatable for better detection, even in cases of low parasitemia, it is essential to carefully define the study sample in order to improve the chances of detecting pathogens present in the study area. Therefore, studies on tick-borne infections in the human population should consider other clinical signs when defining the study sample. It would also be necessary and beneficial to establish a sample from a population at risk of tick bites or in contact with wildlife or domestic ruminants, which would provide valuable information for the epidemiology of tick-borne infections in humans. Populations of farmers, shepherds, slaughterhouse workers, and veterinarians would thus be ideal groups to constitute such a sample.

### 4.2. Prevalent Pathogens, Vectors and Implications for Further Research and Livestock Farming and Human Health

This review’s analysis of the prevalence and distribution of tick-borne pathogens shows that the region of SSA is confronted with numerous challenges in animal and human health. This region is the reservoir for a broad range of pathogens that affect livestock, ticks, and humans. From the above results, it is evident that the prevalence of pathogens varied significantly between the three population under investigation. Humans had a limited array of pathogens detected, with *A. phagocytophilum* and *C. burnetii* being the most frequently reported. In contrast, animals had a larger breadth of infections, with the species of family Anaplasmataceae, i.e., *A. marginale*, *E. ruminantium*, *A. centrale*, as well as *Babesia* spp., i.e., *B. bigemina*, and *B. bovis*, being the most detected. Ticks, on the other hand, harbour a fascinating array of pathogens, including zoonotic ones, the most frequent reported being *E. ruminantium*, *A. marginale*, and *R. africae*. Below is a detailed discussion of all the most frequently identified species of veterinary importance as well as all zoonotic pathogens and their distribution.

*E. ruminantium* is an obligate intracellular rickettsia responsible for heartwater [54]. The disease is responsible for the greatest loss in breeding exotic ruminants and small native ruminants in endemic regions [55]. Transmitted by the ticks of the genus *Amblyomma* spp., this disease has been the subject of numerous studies due to its impact, prevalence, and wide distribution. This explains the frequency of research aimed at detecting the presence of its causal pathogen in both ticks and host animals. Furthermore, this review highlights the potential involvement of ticks *R. decoloratus* and *H. truncatum* (both widespread in SSA) in the epidemiology of heartwater disease [56]. These findings, combined with those already obtained for ticks *R. evertsi* and *H. marginatum* [56], as well as the observed transmission by *R. microplus* [57], an invasive species resistant to acaricides [58,59], pose new challenges for livestock farming in SSA. Moreover, they emphasize the urgent need for in-depth research to better understand and manage the spread of heartwater disease and prevent the emergence of *E. ruminantium* in order to ensure animal health as well as food security in the region [60]. Regarding the difference observed in the frequency of studies that have searched for *E. ruminantium* in animals and ticks, this can be explained by two arguments. Firstly, it can be attributed to abundant documentation on the distribution of its main vectors, such as *A. variegatum* and *A. hebraeum* [61], whose presence is closely related to *E. ruminantium* infection. Secondly, animals act as definitive hosts and are often early indicators of the presence of the disease. This explains why researchers focus on monitoring in order to investigate possibilities of the disease occurrence among animal populations and to identify the significance of large ruminants, suspected to be natural reservoir of *E. ruminantium* [62,63].

*A. marginale* is largely responsible for bovine anaplasmosis, another of the most common blood infections in the whole world, occurring at an endemic level in tropical and subtropical regions [64]. The disease also greatly affects the cattle farm economy, mainly by lowering performance and increasing the mortality rate among animal populations infected with this pathogenic agent [65]. Infection by *A. marginale* has been reported in 18 countries, with 30 studies detecting it in animals compared to 10 studies in ticks. This disparity, as highlighted by Adjou Moumouni et al. (2018), underscores the underutilization of ticks in studies assessing the distribution of livestock diseases in Africa. Although the disease is traditionally transmitted by the tick *H. marginatum rufipes* and *Rhipicephalus* genera ticks, including *R. decoloratus* and *R. microplus* [19,66,67], the *Amblyomma* genus has also raised suspicions for its potential role in the spread of bovine anaplasmosis in SSA. Recent research studies have conducted DNA detection of *A. marginale* in *A. variegatum*-collected ticks in Benin [68], Ethiopia [69], and Madagascar [70], as well as in *A. lepidium* ticks [71] and *A. cohaerens* ticks [72] from Ethiopia. Such possible involvement of *Amblyomma* ticks might have devastating consequences concerning livestock farming in SSA given that *Amblyomma* species are widely distributed in the region. However, studies on the ability of ticks of this genus, particularly *A. variegatum*, to transmit *A. marginale* are essential to define appropriate control measures.

*A. centrale*, once considered a non-pathogenic variant of *A. marginale*, is officially classified as a distinct organism [73]. Unlike its pathogenic counterpart, *A. centrale* is associated with subclinical forms of bovine anaplasmosis, which has led to its use as a live vaccine to protect animals against severe infections caused by *A. marginale* [64]. Just like with *A. marginale*, studies in SSA have focused on identifying *A. centrale* in animals, mainly livestock, rather than in ticks. Thus, eleven studies reported its occurrence in animals, while only two studies reported it in ticks. It is also worth noting the wide geographic distribution of *A. centrale*, reported in 13 countries of SSA, as well as its presence in *A. variegatum* ticks. This widespread distribution of *A. centrale*, coupled with its potential as a live vaccine, holds promising prospects for the management of *A. marginale* infections, which remain a major challenge in the region.

*A. ovis* is a bacterium responsible for anaplasmosis in small ruminants such as sheep and goats. The pathogen has a significant economic impact on the livestock industry worldwide, especially in hot and arid areas or where ectoparasites are prevalent [74]. As highlight by Diarra et al. (2023), none of the studies included in this review investigated its DNA in ticks from West Africa. However, it has been reported in ticks from South Africa, Ethiopia, and Zambia, where it was screened in the *Rhipicephalus* tick and in animals in South [75,76,77,78] and East Africa [79]. Furthermore, both studies that investigated its DNA in sheep in Senegal reported its presence [80,81]. These observations, combined with the widespread and invasive presence of its vectors *Rhipicephalus* [69,82,83,84,85,86,87,88] and *Amblyomma* [85,89], confirm its presence in West Africa. Its non-detection in ticks from this region is therefore attributed to the lack of research on its identification in ticks. Since ticks serve as vectors for this pathogen, any control method should focus on combined data regarding its vector spectrum, the epidemiological role of these vectors in disease transmission, and its prevalence or occurrence in animals.

Bovine babesiosis, a major infectious disease in cattle in Africa, is primarily caused by the species *B. bovis* and *B. bigemina*. This disease can lead to mortality rates of up to 80% in exotic breeds [90]. Studies have revealed that *B. bovis*, due to its increased virulence, is responsible for losses up to 20 times higher than those caused by *B. bigemina*, the indigenous species [91]. However, despite their significant impact on animal health and livestock productivity, an underutilization of ticks in surveillance studies of this disease has been evident. *B. bigemina* has been investigated in twenty-four studies in animals compared to only eight studies in ticks. Similarly, *B. bovis* has been studied in animals in twenty-one studies, while only four studies have focused on ticks. This disparity in the scientific literature raises questions about the effectiveness of disease control strategies, which may not fully consider the role of vectors. *R. annulatus* and *R. microplus* ticks are recognized as the main vectors of *B. bovis* and *B. bigemina*. However, no study has reported the presence of these parasites within these two tick species. Among tick studies, only *R. annulatus* has been used in two studies to investigate *B. bigemina*, and in one study to investigate *B. bovis*. Nevertheless, these pathogens are reported in 15 SSA countries, highlighting that their wide distribution that can only be facilitated by tick vectors. Therefore, further research is necessary to deepen our understanding of the transmission dynamics of bovine babesiosis, with a particular emphasis on the role of tick vectors.

The genus *Theileria* is an haemoprotozoan transmitted by ticks, infecting both domestic and wild animals’ leukocytes and erythrocytes, causing bovine tropical theileriosis [92]. Along with the genus *Anaplasma*, it represents one of the largest genera covered by the studies included in this review, suggesting a high species diversity in the region. The *Theileria* species discussed in many of these studies are *T. parva*, *T. mutans*, *T. velifera*, *T. annulata*, *T. ovis*, and *T. taurotragi*. *T. mutans*, *T. velifera*, and *T. annulata* are ones that are most commonly related to ticks, and while *T. parva* is the one most described in animals, it is followed by those species cited above. The detection frequency of each species within each target population is strongly correlated with the frequency of studies that investigated them. These results once again highlight the neglect of tick studies concerning animal diseases and provide insights into research approaches on TBDs of veterinary importance in SSA. Indeed, research on disease prevalence in hosts takes precedence over that on pathogen prevalence in vectors, which are the primary actors in the distribution of these pathogens [93].

*T. parva*, most frequently reported in studies on theileriosis, is the causative agent of East Coast fever. This bovine disease is the most important theileriosis in SSA, leading to severe economic losses exceeding USD 300 million and mortality exceeding one million in family and nomadic herds [94]. It is widely distributed in East Africa, a region with a high diversity of tick species, where its distribution coincides with the infestation rate of its main vectors, *R. appendiculatus* and *R. zambeziensis* [20]. No studies have reported its presence in West Africa [21]. The three studies reported from this region and included in this review only searched for it in cattle blood. However, studies conducted by Byamukama et al., (2021)**,** in Uganda have shown that some tick species widely distributed in West Africa are capable of carrying *T. parva* [95]. Indeed, its prevalence was reported at 25%, 40%, and 25%, respectively, in the ticks A. variegatum, *R. decoloratus*, and *H. truncatum* [89]. These observations pose threats to West African livestock, extensive and highly exposed to wildlife, as the reservoirs of tick-borne infectious agents. Even though the main tick species that transmit *T. parva* are not present in West Africa, it is possible that tick species colonizing West Africa are capable of acquiring it but incapable of transmitting it. It would be wise for research in this region to focus on the prevalence of this pathogen in ticks to consider subsequent control measures in case of proven transmission of the pathogen by these tick species or in case of an emergence that could lead to the capacity of transmitting this pathogen by these ticks. The other most reported species of *Theileria*, namely *T. mutans*, *T. velifera*, and *T. taurotragi*, are responsible for benign infections [20,96,97]. Although it has been reported that these parasites seem to offer some protection against the pathogenicity of *T. parva* [98], a fatal infection caused by *T. mutans* in an animal previously infected with and immune to *T. parva* has been reported in Kenya [99,100,101]. Indeed, their presence can still have consequences on animal health and welfare, as well as on livestock productivity. Therefore, it would be important and wise to closely monitor the emergence and prevalence of these species, both in ticks and animals, especially in regions where they are present. This would help to detect potential changes in their behaviour or virulence as well as prevent possible outbreaks or economic losses in livestock.

The others interesting result from this review is the presence of various tick-borne viruses, with only one found in animals and none in humans. Among the identified viruses, the Crimean-Congo haemorrhagic fever virus (CCHFV) was the most frequently reported, mentioned in six studies as being present in ticks. CCHFV, an RNA virus belonging to the *Bunyaviridae* family, is transmitted by infected ticks mainly of the genus *Hyalomma* [102], which are widely distributed in Africa [20]. It is known to cause severe haemorrhagic fever in humans. Despite its detection in ticks in the studies included in this review, its absence in animals (potential reservoirs) and humans raises questions regarding the potential for transmission and the true prevalence of this disease among human populations in SSA. Furthermore, this could be attributed to a limited interest among researchers in screening it within the affected populations. Although this virus has generated more research interest than any other virus, only three of the studies included in this review searched for its DNA in humans. Two of these studies were conducted in Senegal, while only one study was carried out in Kenya. It is also important to note that only one of the studies concerned animal subjects. In addition, current studies also highlight the presence of viruses from the Flaviviridae, Nairoviridae, Peribunyaviridae, Poxviridae, and Togaviridae families. Their existence is not as pronounced as that of CCHFV. However, their detection demonstrates the variety of tick virus pathogens present in SSA. Moreover, the detection of viruses such as the Kaptombes virus in animals emphasizes the risk of transmission posed by these pathogens. These observations underscore the need to intensify research on these viral agents to better assess the risk to human health and implement appropriate preventive measures.

### 4.3. Tick-Borne Zoonotic Pathogens in Sub-Saharan Africa and the Need for Integrated One Health Approaches

*Rickettsia africae* is a tick-borne pathogen within the spotted fever group (SFG) of Rickettsiae, known to be transmitted primarily by ticks, particularly *A. variegatum* and *A. hebraeum* [103]. This bacterium is responsible for causing African tick-bite fever in humans, characterized by acute febrile illness accompanied by symptoms such as headache, chills, muscle aches and occasionally a rash mainly observed in tourists traveling through endemic areas [104]. Despite its clinical significance, the dynamics of transmission and the potential vectors involved remain incompletely understood [105]. Our review highlights *R. africae* as the most commonly detected and distributed zoonotic bacteria among those studied. While various tick genera, including *Amblyomma*, *Hyalomma*, and *Rhipicephalus*, have been implicated as potential vectors, no study has conclusively demonstrated the vector competence of *Hyalomma* and *Rhipicephalus* for *R. africae* [21]. Furthermore, the tick samples analyzed in the reviewed studies were predominantly collected from livestock, raising the possibility that the detected DNA originates from the blood of animals rather than tick saliva. In light of the known reservoir role of livestock for *R. africae*, as demonstrated in previous research [106], it is imperative to investigate the potential contribution of livestock to the maintenance and transmission of this pathogen further. However, only one study included in our review specifically searched for *R. africae* DNA in livestock. Therefore, further research, including surveillance for *R. africae* in livestock populations, is warranted to elucidate its role in the epidemiology of African tick-bite fever. Moreover, while *R. africae* has been predominantly studied in ticks, limited attention has been given to its detection in humans. This contrasts with the broader understanding of other rickettsial species, such as *R. bellii*, which have been reported in both humans and animals, suggesting zoonotic transmission of *Rickettsiae* in the region. Therefore, future studies should prioritize investigating the prevalence of Rickettsia species in human populations, applying a “One Health” approach to better understand the dynamics of zoonotic transmission. Additionally, our review identified other rickettsial species within the Rickettsiaceae family, such as *R. sibirica* in *H. truncatum* and *R. massimilae* in *Rhipicephalus* ticks, including *R. senegalensis*, *R. turanicus*, *R. sanguineus*, and *R. lunulatus*. The detection of these species highlights the potential role of these tick species as vectors and the importance of understanding the ecological niches and transmission dynamics associated with these tick species. Further investigation is warranted to determine their prevalence and potential for transmission to humans and animals, with a focus on specific tick vectors identified in this review.

Considering the family of Anaplasmataceae, these studies highlight the presence of zoonotic agents such as *A. capra*, *A. platys-Like*, *A. phagocytophilum*, *E. chaffeensis*, and *E. canis*. For instance, *A. phagocytophilum*, known to be responsible for human and animal granulocytic anaplasmosis [107], has been detected in different *Amblyomma* species, including *A. variegatum*, *A. Lepidium*, and *A. coharensis*. This is concerning given the wide distribution of *Amblyomma* ticks in SSA and their ability to infest various hosts, including livestock, sheep, and humans [20]. Although the epidemiological role of these tick species and other African tick species in which this pathogen has been detected has not been specified [108,109,110], its presence in animals and humans confirms its zoonotic potential. This also suggests the involvement of tick species present in this region of Africa in the transmission and epidemiology of this zoonotic agent. This suggestion is supported by the spatial similarity observed in this study between its presence in ticks and animals. Additionally, the detection of *A. capra*, a newly emerging agent discovered in China [111,112], in ticks in Africa, combined with the presence of other Anaplasmataceae such as *A. platys*, *E. chaffeensis*, and *E. canis* in both ticks and animals, highlights the rapid global dispersion of these pathogens. This also raises concerns about their circulation within livestock in SSA, thereby exposing farmers and travellers in this region to an increased risk. Given that SSA is home to numerous tick species, biosafety measures must be seriously considered to limit the dissemination or introduction of pathogens, as was the case with *R. microplus* and *B. bovis* [19].

*Coxiella burnetii*, the causative agent of Q fever, emerged as the most prevalent bacterium studied in humans. This suggests that *C. burnetii* poses a significant public health concern due to its potential to cause disease in humans. The fact that it has been reported in ticks, animals, and humans in multiple SSA countries further emphasizes its wide distribution and the need for further research into its transmission dynamics and prevention strategies.

In addition, the characterization of zoonotic pathogens within the *Borrelia* genus and *Babesia* species suggests complex dynamics of vector-borne diseases in SSA and the need for integrated surveillance and control strategies to mitigate the risk of zoonotic transmission. These findings further underscore the importance of One Health approaches, which recognize the interconnections between human, animal, and environmental health, and facilitate interdisciplinary collaboration, including between health sectors, veterinary services, and environmental agencies, to address zoonotic disease emergence and spread in SSA.

### 4.4. Detection of Atypical Pathogens for Sub-Sahara Africa

During the period covered by this review, the use of advanced molecular tools associated with sequencing allowed the detection of some pathogens which have never been suspected circulating in SSA. However, in almost all the cases, the detection of such pathogens raised significant epidemiological questions, given the absence or rarity of the usual vectors. *A. phagocytophilum*, *B. microti*, *Ehrlichia muris*, and *R. sibirica* represent good examples of this list of atypical tick-borne pathogens of SSA reported by some studies. They used to be transmitted by *Ixodes* spp. and *Dermacentor* spp. ticks, which are absent or rare in SSA [92,113,114,115,116,117,118,119]. Two main hypotheses could support these findings, although in most of the cases the sequences of the genes studied have not been submitted on GenBank [69,72,120,121,122].

Firstly, migratory birds could allow the transport of infected ticks in SSA which were able to feed on animals in SSA and thus transmitted the pathogens. However, since the tick vectors are absent, this hypothesis supposes that they did arrive to establish in SSA. For instance, among the numerous studies highlighting the capacity of migratory birds to disseminate ticks and the pathogens they transmit [123,124,125,126,127,128], those by Pascucci et al. (2019) and Mancuso et al. (2022) are particularly notable. These studies demonstrated that trans-Saharan migratory birds are capable of carrying African tick species and *Rickettsia*, as well as viruses of zoonotic importance, from Africa into Europe [126,128]. Although these studies focused on northward migration, they illustrate the principle of long-distance tick transport by birds, which could potentially occur in both directions. Thus, similar migratory patterns could facilitate the introduction of these vectors into SSA, where environmental conditions might allow for their establishment and the subsequent transmission of pathogens to local wildlife and livestock [129].

Secondly, the import of foreign races of animals by some modern livestock keepers associated with the not yet known vector competence of local tick species could also lead to such results. In fact, the co-circulation of ticks with some pathogens associated with some environmental parameters (not specifically mastered) can induce the acquirement of vector competence. For instance, in West Africa, following its introduction in 2002–2004 [130,131], it has been recently demonstrated that the invasive tick *Rhipicephalus microplus* has acquired the potential to acquire and transmit *Ehrlichia ruminantium* [57,132].

Obviously, the main limit in some detection of atypical pathogens for SSA is an absence of the sequences of the studied genes in GenBank. Efforts should be made in further investigations to fill this gap.

### 4.5. Study Limitations Induce Possible Underestimation of the Number of Pathogens Present

Our study primarily relied on molecular detection methods for identifying pathogens, despite the existence of a positive correlation between the targeted pathogens and those detected, particularly in ticks and animals. For instance, these techniques, while highly specific and sensitive when properly applied, can sometimes have limited yield [133,134,135,136]. This limitation can be attributed to various factors, such as the quality of the sample and its conservation, the timing of sample collection relative to infection, the pathogen load present, the specificity of the primers used, or the choice of reference loci to target [137,138]. Such limitations may potentially lead to an underestimation of the actual prevalence of the pathogens studied.

To overcome these challenges and achieve a more accurate representation of the epidemiological reality, it is strongly recommended that future studies adopt a diversified diagnostic strategy. This approach should integrate not only conventional molecular methods but also the genomic approaches [134,136,139]. In cases where genomic approaches are not feasible due to their high cost or the technical expertise required for interpreting results [137], conventional molecular methods should be followed by a more in-depth approach. For instance, after amplifying relevant loci, sequencing of these amplicons should be carried out [34,134,136]. The obtained sequences should then be subjected to a BLAST search in GenBank and, in some cases, a phylogenetic analysis for accurate pathogen classification [139]. A precise definition of the population to be sampled, the type and quality of the samples, and consideration of clinical and epidemiological data, where available, would provide more accurate context for the investigation and more in-depth interpretation of the results.

The integration of these multiple data sources and diagnostic methods will offer a more robust and comprehensive estimate of the pathogen load in the populations studied. This global approach will obviously improve the accuracy of prevalence estimates but also contribute to the better understanding of transmission dynamics and the actual impact of these pathogens on animal and human health in the affected regions.

## 5. Conclusions

This systematic study highlights the variety of pathogens transmitted by ticks in SSA, underlining that it is of paramount importance to investigate and monitor them as most of them are zoonotic or potentially zoonotic. While research on bacteria and parasites is progressing, studies on viruses remain largely insufficient. Furthermore, for the pathogens studied, the focus population was ticks for bacteria, and livestock, especially cattle, for parasites. No attention was given to human beings. Therefore, it is essential to deploy further efforts to fill these gaps. This includes exploring zoonotic pathogens and assessing their impact on both human and animal health, investigating the risk factors associated with their emergence, and gathering insights from at-risk populations regarding related biosecurity measures. By shedding light on these dangers and proposing research topics for future works, this study hopes to contribute to the control of TBDs in an approach that addresses both human and animal health as well as the ecosystem where they are.

## Figures and Tables

**Table 1:** Search string used in selected database.

Data Base	Search String
PubMed	“africa south of the sahara”[MeSH Terms] AND (“Tick”[Title/Abstract] OR “Cattle”[Title/Abstract] OR “Goat”[Title/Abstract] OR “Sheep”[Title/Abstract] OR “Human”[Title/Abstract]) AND (“anaplasma”[Title/Abstract] OR “rickettsia”[Title/Abstract] OR “ehrlichia”[Title/Abstract] OR “coxiella”[Title/Abstract] OR “wolbachia”[Title/Abstract] OR “babesia”[Title/Abstract] OR “borrelia”[Title/Abstract] OR “theileria”[Title/Abstract] OR “arboviruses”[MeSH Terms]) AND (2012:2023[pdat])
Scopus	TITLE-ABS-KEY (africa AND NOT (algeria OR egypt OR libya OR morocco OR tunisia)) AND (tick OR cattle OR sheep OR goat) AND (anaplasma OR ehrlichia OR rickettsia OR coxiella OR wolbachia OR babesia OR borrelia OR theileria OR virus OR arbovirus OR “tick-borne virus”)) AND PUBYEAR > 2011 AND PUBYEAR < 2024 AND (LIMIT-TO (DOCTYPE, “ar”))
	TITLE-ABS-KEY (africa AND NOT (algeria OR egypt OR libya OR morocco OR tunisia)) AND human AND (anaplasma OR ehrlichia OR rickettsia OR coxiella OR wolbachia OR babesia OR borrelia OR theileria OR arbovirus OR “tick-borne virus”)) AND PUBYEAR > 2011 AND PUBYEAR < 2024 AND (LIMIT-TO (DOCTYPE, “ar”))
Science Direct	Year: 2012–2023 Title, abstract, keywords: africa AND (tick OR human OR cattle OR sheep OR goat) AND (anaplasma OR rickettsia OR ehrlichia)
	Year: 2012–2023 Title, abstract, keywords: africa AND (tick OR human OR cattle OR sheep OR goat) AND (coxiella OR wolbachia OR borrelia)
	Year: 2012–2023 Title, abstract, keywords: africa AND (tick OR human OR cattle OR sheep OR goat) AND (babesia OR theileria)
	Year: 2012–2023 Title, abstract, keywords: africa AND tick (human OR cattle OR sheep OR goat) AND (virus OR abovirus)

## Data Availability

All relevant data are available in this paper.

## Supplementary Materials

The following supporting information can be downloaded at: https://www.mdpi.com/article/10.3390/pathogens13080697/s1, Table S1: PRISMA 2020 Checklist, File S1: Excel file with raw data.

## Appendix A

**Table A1:** Number of Studies Related to Each Tick-Borne Pathogen Species in Ticks, Animals, and Humans; Their Distribution and Target Loci Used.

Domain	Family	Genus	Species	Tick		Animal		Human		Countries	Target Loci	References
				Screened	Detected	Screened	Detected	Screened	Detected			
	Anaplasmatacea	*Anaplasma*	*Ca. Anaplasma ivorensis*	1	1	NA	NA	NA	NA	Ivory_Coast	TtAna	[140]
			*Ca. Anaplasma africae*	NA	NA	1	1	NA	NA	Senegal	rpoB	[80]
			*A. bovis*	1	1	5	5	NA	NA	Kenya,	16S, groEL	[141,142]
										Malawi,		[143]
										South_Africa,		[144]
										Uganda,		[122]
										Zambia,		[145]
			*A. capra*	1	1	NA	NA	NA	NA	Ghana	16S	[146] or [147]
			*A. centrale*	3	3	11	11	NA	NA	Benin,	16S, groEL, msp2,msp4, rpoB, TtAna	[148]
										Burkina_Faso,		[148]
										Cameroon,		[149]
										Ethiopia,		[71,148]
										Ghana,		[148]
										Ivory_Coast,		[140]
										Kenya,		[150,151]
										Nigeria,		[148,152]
										Senegal,		[80]
										South_Africa,		[73,153]
										Sudan,		[154]
										Tanzania,		[148]
										Uganda,		[122,148,155,156]
										Zambia,		[145]
			*A. marginale*	10	10	32	31	NA	NA	Benin,	16S, msp1, msp2, msp4, msp5, groEL, rpoB, TtAna, MAR1bB2, pCS20,	[16,24,43,68,148,157]
										Botswana		[75]
										Burkina_Faso,		[16,24,148]
										Cameroon,		[149]
										Ethiopia,		[71,72,77,78,148,158]
										Ghana,		[146,147,148,159]
										Guinea,		[160]
										Ivory_Coast,		[140]
										Kenya,		[141,142,150,151,161,162]
										Madagascar,		[70]
										Malawi,		[143]
										Mozambique,		[163]
										Nigeria,		[148,152,164,165,166]
										Senegal,		[80]
										South_Africa,		[73,144,153,167]
										Sudan,		[154,168]
										Tanzania,		[148,169,170]
										Uganda,		[95,122,148,155,171,172]
										Zambia,		[145]
			*A. ovis*	3	3	7	7	NA	NA	Botswana,	16S, msp4, groEL, rpoB,	[75]
										Ethiopia,		[88]
										Kenya,		[79]
										Malawi,		[76]
										Mozambique,		[84]
										Senegal,		[80,81]
										South_Africa,		[144,173]
										Sudan		[154]
			*A. phagocytophilum*	3	3	5	4	1	1	Ethiopia,	16S, msp2, groEL	[71,72]
										South_Africa,		[34,173,174]
										Senegal,		[81]
										Mozambique,		[163]
										Uganda,		[122]
			*A. platys*	1	1	1	1	NA	NA	Senegal,	16S, groEL	[81]
										South_Africa,		[144]
			*A. platys-like*	1	1	9	9	NA	NA	Cameroon,	16S, 18S, groEL, msp4, rpoB,	[149]
										Guinea,		[160]
										Kenya,		[141,142,150,151,162]
										Malawi,		[143]
										Nigeria,		[165]
										Senegal,		[80]
										Uganda,		[95]
			*Anaplasma* sp. *Dedessa*	NA	NA	1	1	NA	NA	Ethiopia,	16S	[158]
			*Anaplasma* sp. *Hadesa*	NA	NA	2	2	NA	NA	Cameroon,	16S	[149]
										Ethiopia,		[158]
			*Anaplasma* sp. *Lambwe-1*	NA	NA	1	1	NA	NA	Kenya,	16S	[141]
			*Anaplasma* sp. *Omatjenne*	1	1	3	3	NA	NA	Ethiopia,	16S	[71,158]
										Uganda,		[122]
										Zambia,		[145]
			*Anaplasma* sp. *Saso*	NA	NA	1	1	NA	NA	Ethiopia,	16S	[158]
			*Anaplasma* spp.	4	3	14	14	1	0	Angola,	16S, groEL, msp4, msp5, TtAna	[175]
										Cameroon,		[149]
										Ethiopia,		[88]
										Ivory_Coast,		[140]
										Kenya,		[79,141,142,162]
										Malawi,		[76,143]
										Mozambique,		[163]
										Nigeria,		[152,165,176]
										Senegal,		[35,81]
										South_Africa,		[177]
										Sudan,		[154]
										Tanzania		[178]
			*Anaplasma/Ehrlichia* spp.	2	1	3	3	NA	NA	Cameroon,	16S	[149]
										Ethiopia,		[88]
										Nigeria,		[152,179]
										South_Africa,		[144]
		*Ehrlichia*	*Ca. Ehrlichia rustica*	1	1	NA	NA	NA	NA	Ivory_Coast,	TtAna	[140]
			*Ca. Ehrlichia urmitei*	1	1	NA	NA	NA	NA	Ivory_Coast,	TtAna	[140]
			*E. canis*	4	4	2	2	NA	NA	Ghana,	16S, dsbA, groEL	[146,147]
										Malawi,		[76]
										Senegal,		[80]
										South_Africa,		[120,180]
										Zambia,		[145]
			*E. chaffeensis*	1	1	1	1	NA	NA	South_Africa,	16S, dsbA	[120]
										Zambia,		[145]
			*E. minasensis*	2	2	3	3	NA	NA	Ethiopia,	16S, dsbA	[158]
										Ghana,		[146,147]
										Kenya,		[142,150,151]
										South_Africa,		[120]
			*E. muris*	1	1	NA	NA	NA	NA	South_Africa,	dsbA	[120]
			*E. ruminantium*	15	15	20	16	NA	NA	Benin,	16S, dsbA, groEL, pCS20, TtAna	[16,24,43,68,148,157]
										Burkina_Faso,		[16,24,148]
										Cameroon,		[149,181,182]
										Ethiopia,		[71,72,77,78,88,148,158]
										Ghana,		[148]
										Ivory_Coast,		[140]
										Kenya,		[79]
										Malawi,		[76]
										Mozambique,		[183]
										Nigeria,		[148,152,184,185]
										Senegal,		[81]
										South_Africa,		[144,167,173,174,180,186,187]
										Tanzania,		[148,170]
										Uganda,		[95,122,148,156]
										Zambia,		[145]
			*Ehrlichia* spp.	8	7	7	4	NA	NA	Angola,	16S, dsbA, groEL, gltA, TtAna	[175]
										Cameroon,		[149]
										Ethiopia,		[88]
										Guinea,		[160]
										Ivory_Coast,		[140]
										Kenya,		[142,150,151,162]
										Malawi,		[143]
										South_Africa,		[120,144,180]
										Sudan,		[154]
										Tanzania,		[178]
	Coxiellacea	*Coxiella*	*C. burnetii*	11	8	2	1	4	2	Angola,	COX, htpB, IS1111	[175]
										Ethiopia,		[188,189]
										Ghana,		[146,147]
										Ivory_Coast,		[140]
										Kenya,		[190]
										Madagascar,		[191]
										Sao_Tome		[192]
										Senegal,		[35,193]
										South_Africa,		[167,174,194,195]
										Zanzibar		[196]
			*Coxiella* spp.	4	3	NA	NA	NA	NA	Angola,	16S, rpoB, groEL	[175]
										Sao_Tome,		[192]
										South_Africa,		[197]
										Tanzania		[178]
	Rickettsiacea	*Rickettsia*	*Ca. Rickettsia barbariae*	1	1	NA	NA	NA	NA	Cameroon	ompB	[121]
			*R. aeschlimannii*	5	5	NA	NA	NA	NA	Angola,	ompA, ompB, RaescSca1	[175]
										Cameroon,		[121]
										Ghana,		[146,147]
										Ivory_Coast,		[140]
										Zambia,		[198]
			*R. africae*	21	21	1	1	1	0	Angola,	16S, gltA, ompA, ompB, 17 kDa, poT15-dam2	[175]
										Burkina_Faso,		[199]
										Cameroon,		[121,149]
										Comoros,		[200]
										Djibouti,		[201]
										Ethiopia,		[88,199,202]
										Ghana,		[146,147]
										Guinea,		[18]
										Ivory_Coast,		[140]
										Kenya,		[31,203]
										Liberia,		[18]
										Madagascar,		[204]
										Mozambique,		[84,205]
										Nigeria,		[206]
										South_Africa,		[105,186,207,208]
										Sudan,		[209]
										Tanzania,		[200]
										Zambia,		[198]
			*R. massiliae*	4	4	NA	NA	NA	NA	Cameroon,	Hypothetical protein, 23S-5S, ompB	[121]
										Ivory_Coast,		[140]
										Nigeria,		[206,210]
			*R. rickettsii*	1	1	NA	NA	NA	NA	South_Africa	16S	[197]
			*R. sibirica*	1	1	NA	NA	NA	NA	Cameroon	ompB	[121]
			*Rickettsia* spp.	27	25	6	2	2	1	Angola,	16S, gltA, ompA, ompB, 17 kDa,	[175]
										Benin,		[43,68,211]
										Burkina_Faso,		[199]
										Cameroon,		[121,149]
										Djibouti,		[201]
										Ethiopia,		[88,188,199,212]
										Ghana,		[146,147]
										Guinea,		[18]
										Ivory_Coast,		[140]
										Kenya,		[141,162,213]
										Liberia,		[18]
										Mozambique,		[84,205]
										Nigeria,		[152,176,206]
										Sao_Tome,		[192]
										Senegal,		[35]
										South_Africa,		[167,173,174,194,208,214,215]
										Sudan,		[209]
										Tanzania,		[178]
										Togo,		[211]
										Uganda,		[46]
										Zambia,		[198]
										Zanzibar		[196]
			*R. felis*	NA	NA	1	1	1	1	Cameroon,	16S	[149]
										Ethiopia,		[216]
			*R. bellii*	NA	NA	NA	NA	1	1	Ethiopia,	16S	[216]
		*Wolbachia*	*Ca. Wolbachia ivorensis*	1	1	NA	NA	NA		Ivory_Coast,	TtAna	[140]
	Spirochaetaceae	*Borrelia*	*Ca. Borrelia africana*	1	1	NA	NA	NA	NA	Ivory_Coast,	Bor ITS4	[140]
			*Ca. Borrelia ivorensis*	1	1	NA	NA	NA	NA	Ivory_Coast,	Bor ITS4	[140]
			*B. burgdorferi*	1	0	NA	NA	NA	NA			[174]
			*B. theileri*	1	1	1	1	NA	NA	Cameroon,	18S, flaB	[149]
										Mali,		[217]
			*Borrelia* spp.	5	3	2	1	2	1	Angola,	16S, flaB, Bor ITS4	[175]
										Cameroon,		[149]
										Ethiopia,		[212,216]
										Ivory_Coast,		[140]
										Madagascar,		[218]
										Tanzania,		[178]
										Uganda		[95]
			*B. crocidurae*	NA	NA	NA	NA	1	1	Senegal,	glpQ	[35]
Parasite	Babesidae	*Babesia*	*B. bigemina*	8	7	24	23	NA	NA	Angola,	16S, 18S, bs1, ITS1, ITS2, ama1, cytb, rap1a, speI_avaI	[175]
										Benin,		[16,24,43,68,148,157]
										Burkina_Faso,		[16,24,148,219]
										Ethiopia,		[77,78,148,158]
										Ghana,		[148,159]
										Guinea,		[160]
										Kenya,		[150,151,161,162,220]
										Lesitho,		[221]
										Malawi,		[143]
										Nigeria,		[148,152,185]
										South_Africa,		[144,167,222,223,224]
										Sudan,		[168]
										Tanzania,		[148,169,170]
										Uganda,		[95,122,148,156,171,172]
										Zambia,		[145]
			*B. bovis*	4	3	21	14	1	NA	Benin,	16S, 18S, BoF2, cytb, rap1, sbp2, sbp4	[16,24,43,68,148,157]
										Burkina_Faso,		[16,24,148,219]
										Ethiopia,		[77,78,148]
										Ghana,		[148,225,226]
										Kenya,		[161,220]
										Lesitho,		[221]
										Mozambique,		[227]
										Nigeria,		[148,152]
										South_Africa,		[167,173,222,223,224,227]
										Sudan,		[168]
										Tanzania,		[148,169,170]
										Uganda,		[95,148,171,172]
										Zambia,		[145]
			*B. caballi*	2	2	1	1	NA	NA	Ethiopia,	18S	[188]
										South_Africa,		[144]
										Zambia,		[145]
			*B. divergens*	NA	NA	1	0	1	NA	Ghana	18S	[225]
			*B. microti*	1	1	NA	NA	NA	NA	Uganda,	18S	[156]
			*B. motasi*	1	1	NA	NA	NA	NA	Lesitho,	NA	[221]
			*B. occultans*	1	1	1	1	NA	NA	Burkina_Faso,	18S	[219]
										South_Africa,		[144]
			*B. ovis*	1	1	1	0	NA	NA	Kenya,	NA	[79]
										Lesitho,		[221]
			*B. rossi*	1	1	NA	NA	NA	NA	Uganda,	18S	[156]
			*Babesia* sp. *sable*	1	1	1	1	NA	NA	South_Africa,	18S	[144]
										Zambia,		[145]
			*Babesia* spp.	2	2	2	0	NA	NA	Angola,	18S, ITS1, ITS2	[175]
										Kenya,		[141]
										Sudan,		[228]
										Tanzania,		[178]
			*B. gibsoni*	NA	NA	2	2	NA	NA	Malawi,	18S	[76]
										Zambia,		[145]
			*B. canis*	NA	NA	NA	NA	1	NA	Ghana	18S	[225]
			*Babesia* sp. *mymensingh*	NA	NA	1	1	NA	NA	Uganda,	ama1	[171,172]
	Theileridae	*Theileria*	*T. orientalis*	2	2	7	4	NA	NA	Benin,	18S, mpsp	[157]
										Burkina_Faso,		[219]
										Ethiopia,		[77,158,188,229,230]
										Kenya,		[161]
										South_Africa,		[231]
			*T. velifera*	5	5	14	14	NA	NA	Benin,	16S, 18S	[16,24]
										Burkina_Faso,		[16,24,219]
										Cameroon,		[149]
										Ethiopia,		[77,158,188,229]
										Kenya,		[141,150,151,161,162]
										Malawi,		[143]
										Mozambique,		[84]
										Nigeria,		[152]
										South_Africa,		[231]
										Sudan,		[232]
										Uganda,		[122,156]
										Zambia,		[145]
			*T. annulata*	5	3	7	5	NA	NA	Benin,	16S, 18S, tams1	[16,24,43,68,157]
										Burkina_Faso,		[16,24,219]
										Ethiopia,		[77,229]
										Guinea,		[160]
										Nigeria,		[233]
										South_Africa,		[144]
										Sudan,		[228,232,234]
			*T. bicornis*	1	1	NA	NA	NA	NA	South_Africa,	18S	[144]
			*T. buffeli*	1	1	1	1	NA	NA	South_Africa,	18S	[144]
										Zambia,		[145]
			*T. mutans*	6	6	19	19	NA	NA	Benin,	16S, 18S	[16,24,43,68,157]
										Burkina_Faso,		[25,26,107]
										Cameroon,		[149,235]
										Ethiopia,		[77,78,158,188,229,236]
										Kenya,		[141,150,151,161]
										Malawi,		[76,143]
										Nigeria,		[152]
										South_Africa,		[144,167]
										Sudan,		[232]
										Tanzania,		[170]
										Uganda,		[122,156]
										Zambia,		[145]
			*T. ovis*	2	1	7	7	NA	NA	Ethiopia,	18S	[229]
										Kenya,		[161]
										Lesitho,		[221]
										Malawi,		[76]
										South_Africa,		[144,173]
										Sudan,		[228,237]
										Tanzania,		[170]
			*T. parva*	2	2	32	30	NA	NA	Benin,	18S, p104, COI	[148,157]
										Burkina_Faso,		[148,219]
										Burundi,		[238,239]
										Cameroon,		[235]
										Congo,		[240]
										Ethiopia,		[77,78,148]
										Ghana,		[148]
										Guinea,		[160]
										Kenya,		[150,151,161,162]
										Lesitho,		[221]
										Malawi,		[143]
										Nigeria,		[148]
										Rwanda,		[241]
										South_Africa,		[167,242]
										Sudan,		[243]
										Tanzania,		[148,169,170,244,245,246,247]
										Uganda,		[95,122,148,156,171,172,248,249,250,251,252]
										Zambia,		[145,253]
			*T. separata*	2	2	4	4	NA	NA	Ethiopia,	18S	[229]
										Malawi,		[76]
										South_Africa,		[144]
										Sudan,		[228,237]
										Uganda,		[156]
			*T. taurotragi*	2	2	12	10	NA	NA	Benin,	18S	[157]
										Burkina_Faso,		[219]
										Ethiopia,		[77,78]
										Kenya,		[150,151,161]
										Malawi,		[143]
										Nigeria,		[152]
										South_Africa,		[144,167]
										Tanzania,		[170,254]
										Uganda,		[122]
										Zambia,		[145]
			*T. equi*	NA	NA	1	1	NA	NA	Zambia,	18S	[145]
			*T. lestoquardi*	NA	NA	4	4	NA	NA	Sudan,	18S, msp	[228,234,237,255]
			*Theileria* sp. *Buffalo*	NA	NA	3	3	NA	NA	Kenya,	18S	[161]
										South_Africa,		[242]
										Zambia,		[145]
			*Theileria* sp. *Kudu*	1	1	1	1	NA	NA	South_Africa,	18S	[144]
										Zambia,		[145]
			*Theileria* sp. *Sable*	1	1	1	1	NA	NA	South_Africa,	18S	[144]
										Zambia,		[145]
			*Theileria* spp.	5	4	10	10	NA	NA	Agola,	18S, cytb	[175]
										Cameroon,		[149]
										Ethiopia,		[229]
										Ghana,		[159]
										Kenya,		[79,141,161]
										Malawi,		[76]
										Nigeria,		[256]
										South_Africa,		[144,231]
										Tanzania,		[170,178]
										Uganda,		[95,156]
	Theileridae/ Babesidae	*Theileria/* *Babesia*	*Theileria/Babesia* spp.	1	1	5	4	1		Cameroon,	18S	[149]
										Ghana,		[225]
										Nigeria,		[176,179]
										South_Africa,		[144]
										Sudan,		[232]
Virus	Alphaviridae	*Alphavirus*	*Alphavirus*	1	0	1	0	NA	NA	Guinea,	L_segment	[257]
										Kenya		[150,151]
	Bunyaviridae	*Phlebovirus*	*Balambala tick virus*	1	1	NA	NA	NA	NA	Ghana,	L_segment	[258]
			BDTPV	1	1	NA	NA	NA	NA	Kenya,	RdRp	[259]
			BOGV	1	1	NA	NA	NA	NA	Kenya,	RdRp	[259]
			*Bole tick virus*	1	1	NA	NA	NA	NA	Kenya,	RdRp	[259]
			PERV	1	1	NA	NA	NA	NA	Kenya,	RdRp	[259]
			*Phlebovirus*	4	1	1	0	NA	NA	Burkina_Faso,	RdRp, N_segment, S_segment	[260]
										Ghana,		[261]
										Guinea,		[257]
										Kenya,		[150,151,259]
			*Phlebovirus DSP4*	1	1	NA	NA	NA	NA	Kenya,	RdRp	[259]
			*Rift Valley Fever Virus*	1	0	NA	NA	NA	NA	Burkina_Faso	G2	[260]
			*Shibuyunji virus*	1	1	NA	NA	NA	NA	Zambia,	L_segment	[262]
	Flaviridae	*Flavivirus*	*Flavivirus*	2	0	1	0	NA	NA	Burkina_Faso,	NS5, L_segment	[260]
										Guinea,		[257]
										Kenya		[150,151]
			JMTV	1	1	NA	NA	NA	NA	Kenya,	NS5	[263]
	Nairoviridae	*Nairovirus*	*Nairovirus*	1	1	1	0	NA	NA	Ghana,	L_segment	[261]
										Kenya		[150,151]
		*Orthonairovirus*	*Orthonairovirus*	1	0	NA	NA	NA	NA	Guinea	NA	[257]
			CCHFV	7	6	1	0	3	0	Burkina_Faso, Cameroon, Kenya, Mauritania, Senegal, South_Africa, Uganda, Zambia,	L_segment, N_segment, S_segment	[260] [264] [31] [265] [32,33] [266] [267]
			DUGV	4	4	NA	NA	NA	NA	Cameroon,	L_segment, S_segment	[264]
										Ghana,		[258,261]
										Nigeria,		[268]
	Peribunyaviridae	*Orthobunyavirus*	*Ngari virus*	1	1	NA	NA	NA	NA	Guinea,	NgvS	[257]
			*Orthobunyavirus*	1	1	1	0	NA	NA	Guinea,	S_segment	[257]
										Kenya		[150,151]
	Poxyviridae	*Parapoxvirus*	*Parapoxvirus*	1	1	NA	NA	NA	NA	Burkina_Faso,	B2L/J6R	[260]
			BPSV	1	1	NA	NA	NA	NA	Burkina_Faso,	BPSV_J6R	[260]
			PCPV	1	1	NA	NA	NA	NA	Burkina_Faso,	PCPV_J6R	[260]
		*Orthopoxvirus*	*Orthopoxvirus*	1	0	NA	NA	NA	NA	Burkina_Faso,	HA(J7R)	[260]
	Togaviridae	*Orbivirus*	*Orbivirus*	1	0	NA	NA	NA	NA	Guinea	NA	[257]
			KPTV	NA	NA	1	1	NA	NA	Kenya	segment 2	[269]
		*Thogotovirus*	*Dhori virus*	NA	NA	1	0	NA	NA	Kenya	S_segment	[150,151]
			*Thogotovirus*	NA	NA	1	0	NA	NA	Keny	M_segment	[150,151]

Legend: Ca: Candidatus, BDTPV: Brown dog tick phlebovirus; BOGV: Bogoria virus; PERV: Perkerra virus; JMTV: Jingmen tick virus; BPSV: Bovine papular stomatitis virus; PCPV: Pseudocowpox virus; KPTV: Kaptombes virus; BTV: Balanbala tick virus; BoTV: Bole tick virus; NA: Not available.
